# Supplementary material for: Gene Expression and Isoform Identification of PacBio Full-Length cDNA Sequences for Berberine Biosynthesis in Berberis koreana
Source: Plants (Basel). 2021 Jun 28;10(7):1314. doi: 10.3390/plants10071314 (PMC8308982; doi:10.3390/plants10071314)
Supplement: Supplementary file 1 [file plants-10-01314-s001.zip › Supple figs.pptx]

## Slide 1
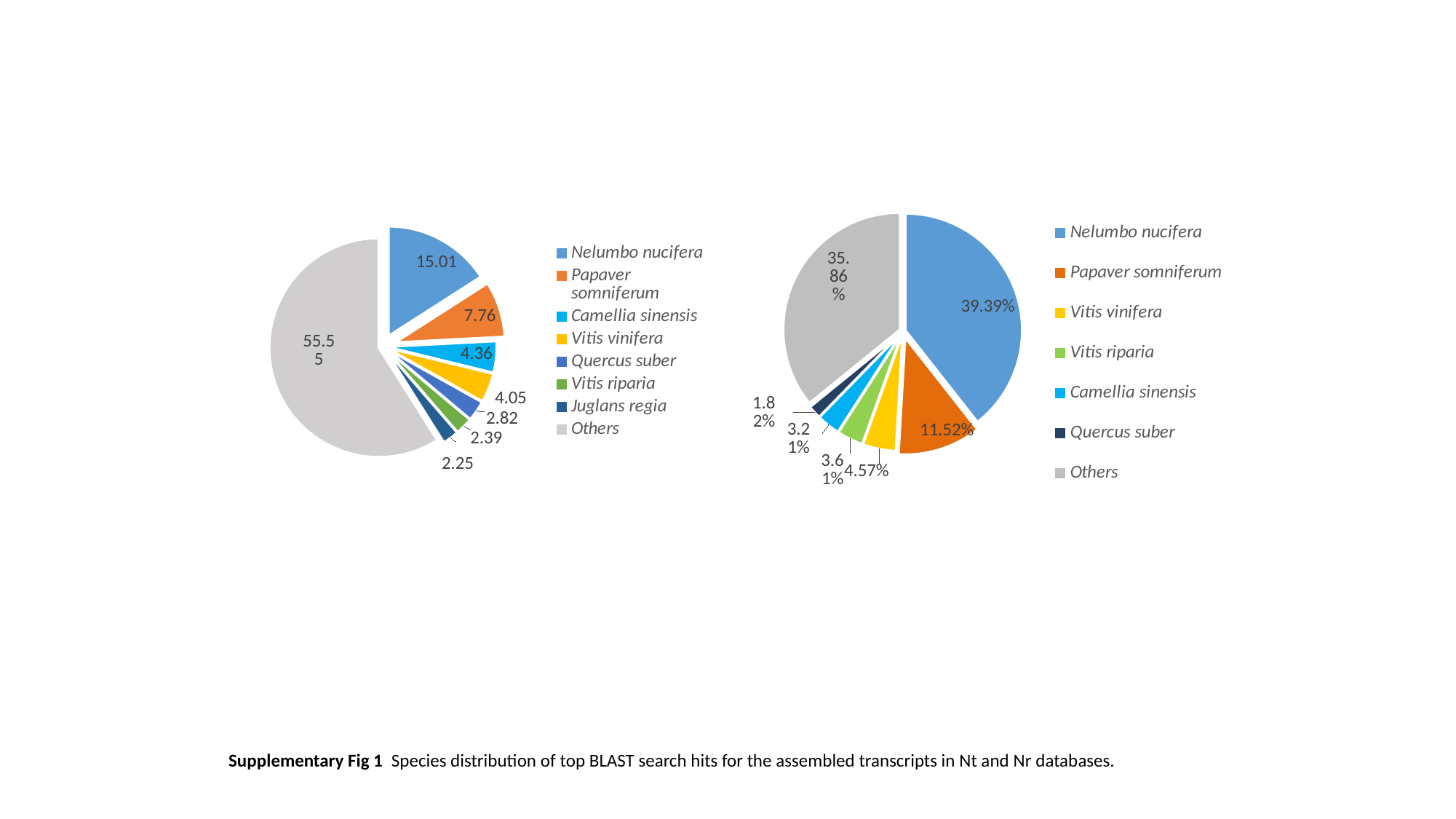

### Chart
| Category | |
|---|---|
| Nelumbo nucifera | 15.009736855245418 |
| Papaver somniferum | 7.75952464173366 |
| Camellia sinensis | 4.359115194487441 |
| Vitis vinifera | 4.054526389374344 |
| Quercus suber | 2.8211913916213116 |
| Vitis riparia | 2.391771109002846 |
| Juglans regia | 2.2519598541968344 |
| Others | 55.55000748988866 |
### Chart
| Category | |
|---|---|
| Nelumbo nucifera | 15082.0 |
| Papaver somniferum | 4412.0 |
| Vitis vinifera | 1750.0 |
| Vitis riparia | 1385.0 |
| Camellia sinensis | 1228.0 |
| Quercus suber | 700.0 |
| Others | 13730.0 |Supplementary Fig 1 Species distribution of top BLAST search hits for the assembled transcripts in Nt and Nr databases.

## Slide 2
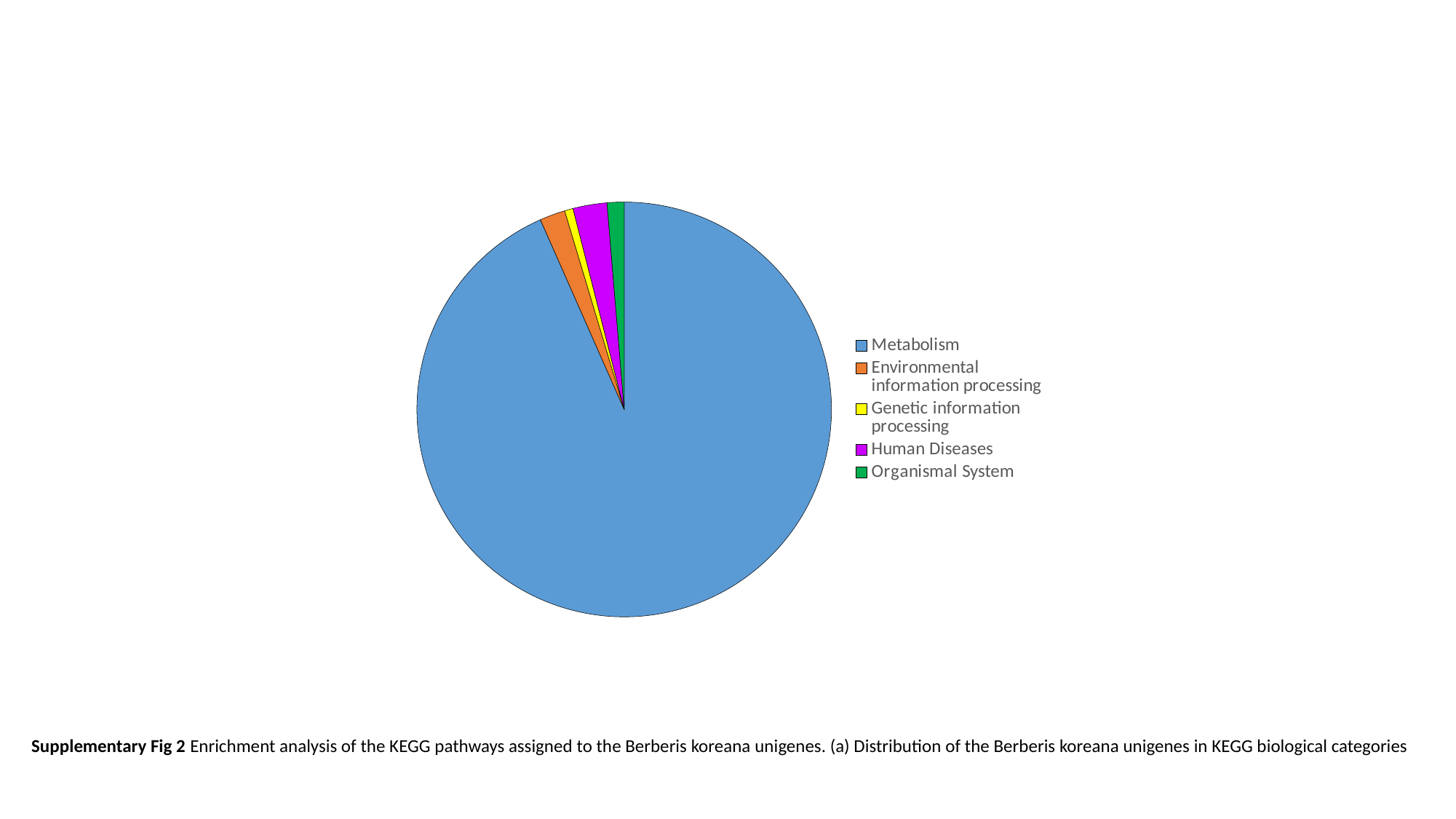

### Chart
| Category | |
|---|---|
| Metabolism | 141.0 |
| Environmental information processing | 3.0 |
| Genetic information processing | 1.0 |
| Human Diseases | 4.0 |
| Organismal System | 2.0 |Supplementary Fig 2 Enrichment analysis of the KEGG pathways assigned to the Berberis koreana unigenes. (a) Distribution of the Berberis koreana unigenes in KEGG biological categories

## Slide 3
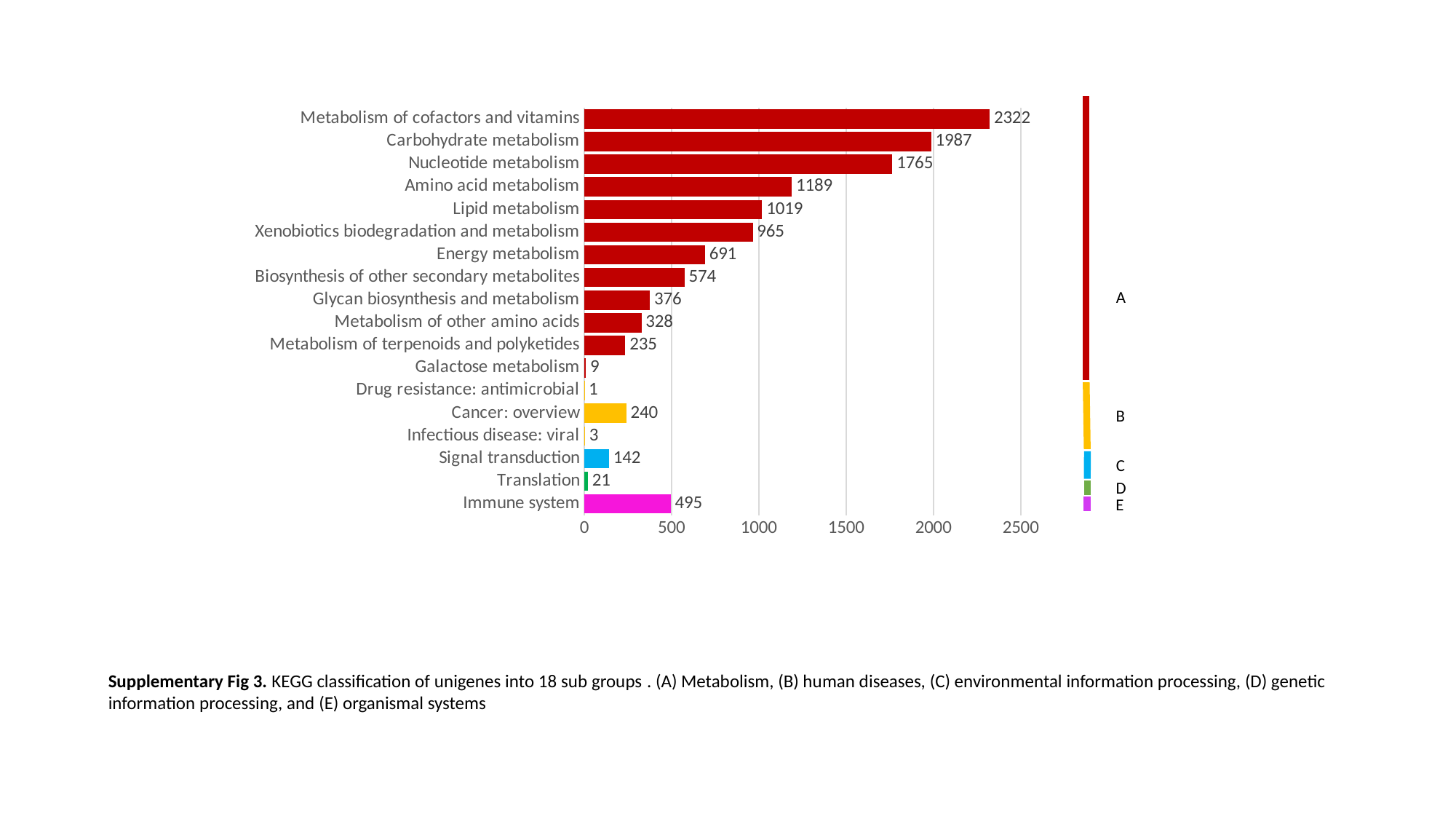

### Chart
| Category | Number of unigenes |
|---|---|
| Immune system | 495.0 |
| Translation | 21.0 |
| Signal transduction | 142.0 |
| Infectious disease: viral | 3.0 |
| Cancer: overview | 240.0 |
| Drug resistance: antimicrobial | 1.0 |
| Galactose metabolism | 9.0 |
| Metabolism of terpenoids and polyketides | 235.0 |
| Metabolism of other amino acids | 328.0 |
| Glycan biosynthesis and metabolism | 376.0 |
| Biosynthesis of other secondary metabolites | 574.0 |
| Energy metabolism | 691.0 |
| Xenobiotics biodegradation and metabolism | 965.0 |
| Lipid metabolism | 1019.0 |
| Amino acid metabolism | 1189.0 |
| Nucleotide metabolism | 1765.0 |
| Carbohydrate metabolism | 1987.0 |
| Metabolism of cofactors and vitamins | 2322.0 |A
B
C
D
E
Supplementary Fig 3. KEGG classification of unigenes into 18 sub groups . (A) Metabolism, (B) human diseases, (C) environmental information processing, (D) genetic information processing, and (E) organismal systems

## Slide 4
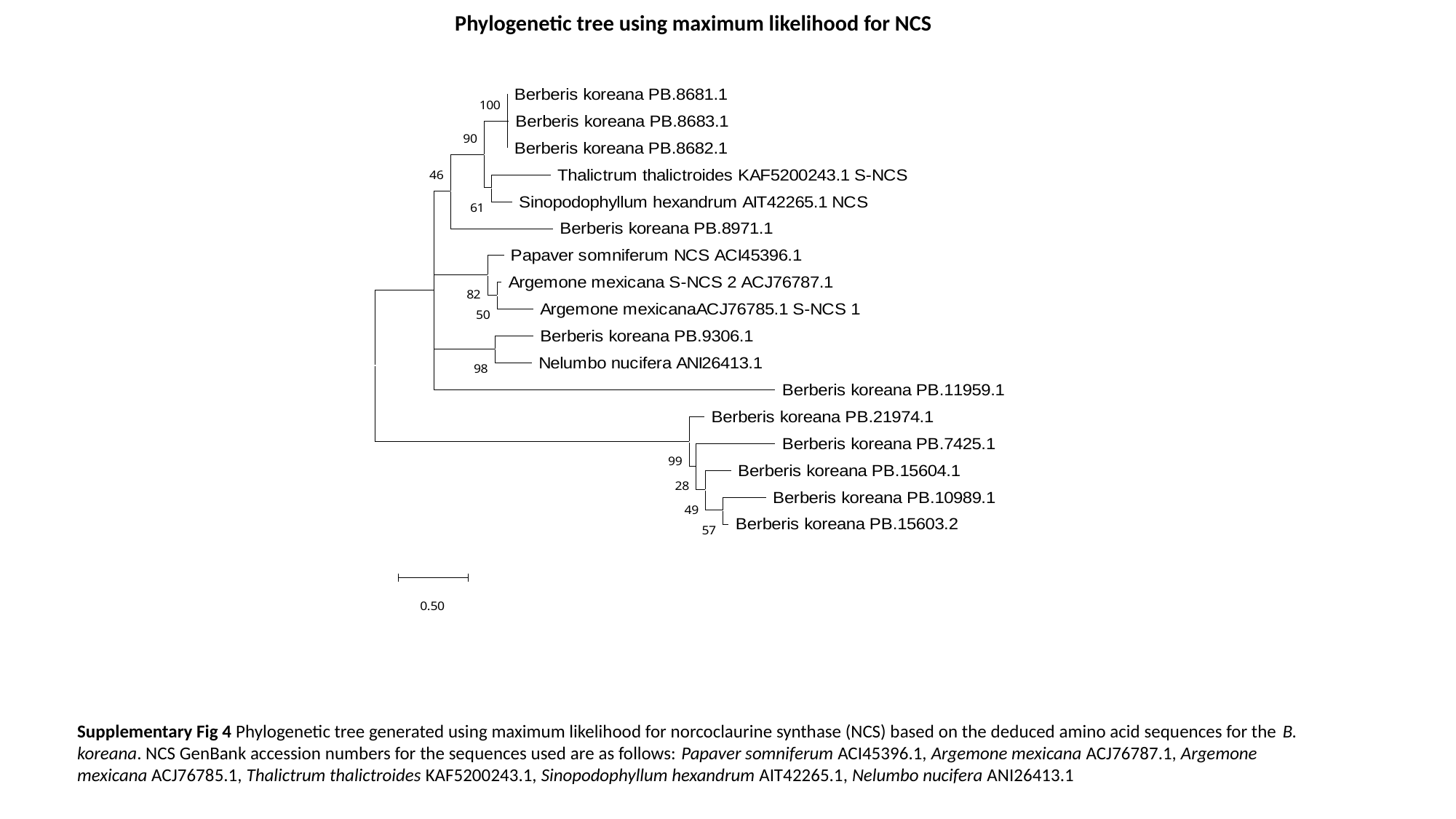

Phylogenetic tree using maximum likelihood for NCS
Supplementary Fig 4 Phylogenetic tree generated using maximum likelihood for norcoclaurine synthase (NCS) based on the deduced amino acid sequences for the B. koreana. NCS GenBank accession numbers for the sequences used are as follows: Papaver somniferum ACI45396.1, Argemone mexicana ACJ76787.1, Argemone mexicana ACJ76785.1, Thalictrum thalictroides KAF5200243.1, Sinopodophyllum hexandrum AIT42265.1, Nelumbo nucifera ANI26413.1

## Slide 5
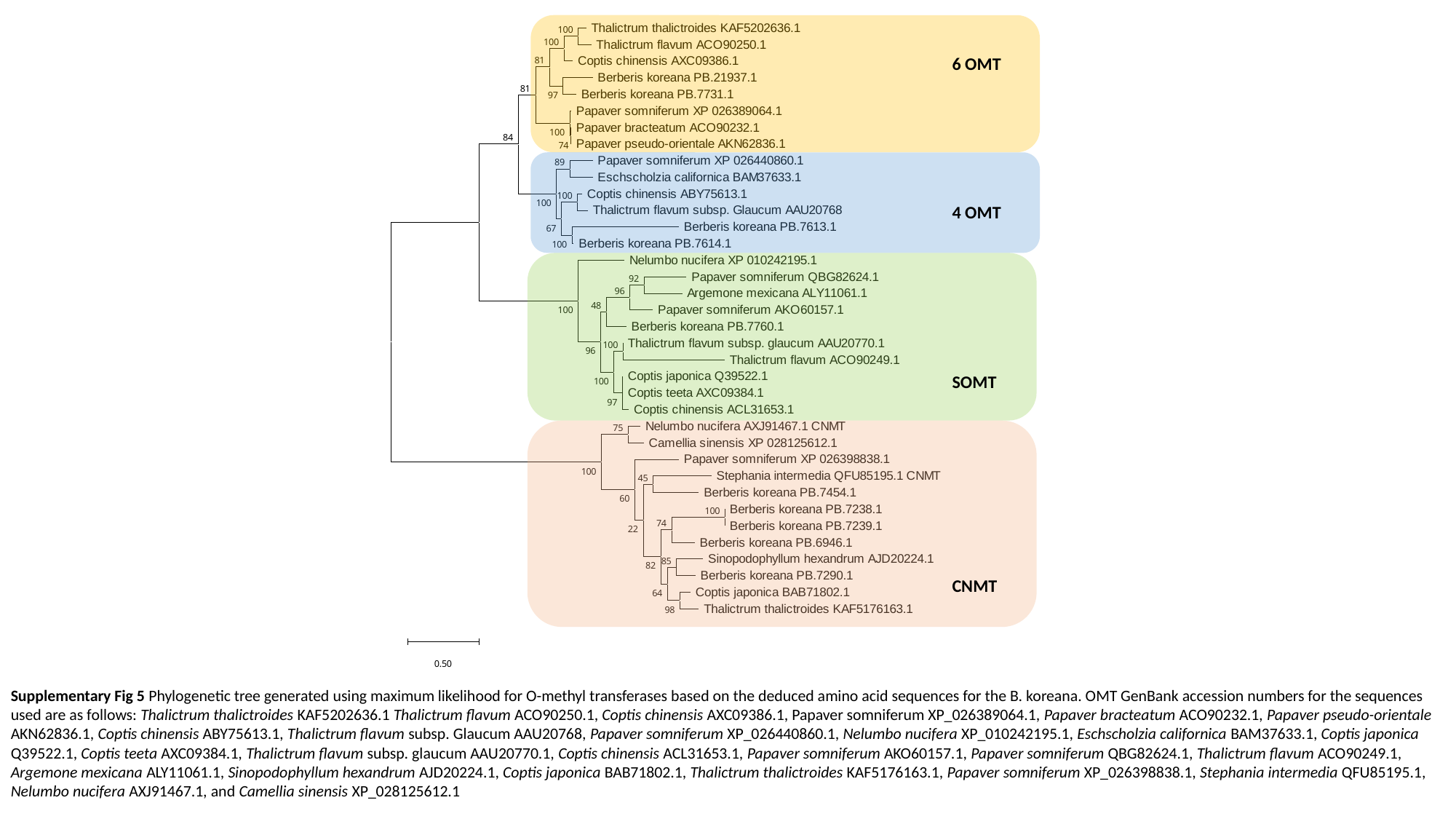

6 OMT
4 OMT
SOMT
CNMT
Supplementary Fig 5 Phylogenetic tree generated using maximum likelihood for O-methyl transferases based on the deduced amino acid sequences for the B. koreana. OMT GenBank accession numbers for the sequences used are as follows: Thalictrum thalictroides KAF5202636.1 Thalictrum flavum ACO90250.1, Coptis chinensis AXC09386.1, Papaver somniferum XP_026389064.1, Papaver bracteatum ACO90232.1, Papaver pseudo-orientale AKN62836.1, Coptis chinensis ABY75613.1, Thalictrum flavum subsp. Glaucum AAU20768, Papaver somniferum XP_026440860.1, Nelumbo nucifera XP_010242195.1, Eschscholzia californica BAM37633.1, Coptis japonica Q39522.1, Coptis teeta AXC09384.1, Thalictrum flavum subsp. glaucum AAU20770.1, Coptis chinensis ACL31653.1, Papaver somniferum AKO60157.1, Papaver somniferum QBG82624.1, Thalictrum flavum ACO90249.1, Argemone mexicana ALY11061.1, Sinopodophyllum hexandrum AJD20224.1, Coptis japonica BAB71802.1, Thalictrum thalictroides KAF5176163.1, Papaver somniferum XP_026398838.1, Stephania intermedia QFU85195.1, Nelumbo nucifera AXJ91467.1, and Camellia sinensis XP_028125612.1
